# Tracking by Identification Using Computer Vision and Radio

**DOI:** 10.3390/s130100241

**Published:** 2012-12-24

**Authors:** Rok Mandeljc, Stanislav Kovačič, Matej Kristan, Janez Perš

**Affiliations:** Machine Vision Laboratory, Faculty of Electrical Engineering, University of Ljubljana, Tržaška 25, SI-1000 Ljubljana, Slovenia; E-Mails: stanislavkovacic@fe.uni-lj.si (S.K.); matej.kristan@fe.uni-lj.si (M.K.); janez.pers@fe.uni-lj.si (J.P.)

**Keywords:** person localization, identification, tracking, radio, computer vision, multi-camera, sensor fusion, tracking-by-identification

## Abstract

We present a novel system for detection, localization and tracking of multiple people, which fuses a multi-view computer vision approach with a radio-based localization system. The proposed fusion combines the best of both worlds, excellent computer-vision-based localization, and strong identity information provided by the radio system, and is therefore able to perform tracking by identification, which makes it impervious to propagated identity switches. We present comprehensive methodology for evaluation of systems that perform person localization in world coordinate system and use it to evaluate the proposed system as well as its components. Experimental results on a challenging indoor dataset, which involves multiple people walking around a realistically cluttered room, confirm that proposed fusion of both systems significantly outperforms its individual components. Compared to the radio-based system, it achieves better localization results, while at the same time it successfully prevents propagation of identity switches that occur in pure computer-vision-based tracking.

## 1. Introduction

In the past two decades, the problem of object detection, localization and tracking received significant attention. This coincides with the rising demand for information about objects' location and identity, which stems from applications in various fields, such as manufacturing, military, surveillance and security, transport and logistics, medical care, childcare, performance analysis in sports and sports medicine. Various localization solutions based on different sensor modalities have been proposed [1], with two most prominent research areas being detection and tracking using video cameras [2] and localization using radio technology [3]. Sensor fusion has also gained prominence as a paradigm for overcoming limitations of individual sensor modalities [1].

The need for recovery of individuals' positions and trajectories, measured in the world coordinate system (*i.e.*, room coordinate system as opposed to an image plane), can be found in different scenarios, most notably in closed-world surveillance, intelligent environments and performance analysis in sports. In surveillance, knowing individuals' position and identity enables us not only to determine their presence or absence, but also analyze their behavior, detect abnormalities in it and reconstruct events. Similarly, in sports, obtaining athletes' trajectories allows a consistent analysis of game measures, such as movement of the individual players or the whole team, physiological demands and intensity of the game, and strategy assessment, which has proven to be very valuable for coaches and sports physiologists. Therefore, a person localization system can be regarded as a measuring device that measures individuals' positions and determines their identities. As can be seen from the short survey on currently-used player tracking techniques in sports [4], attempts have been made to use both radio-based and camera-based approaches.

Both radio and video sensor modality have their advantages and disadvantages, which are often of complementary nature. The main disadvantage of radio-based localization and tracking systems is that they are intrusive; they comprise a network of radio receivers (sensors) and radio-emitting tags that people need to wear. While inconvenient, this is still acceptable in environments where people are already expected to wear identification tags, for example in some surveillance applications and intelligent environments. In sports, however, intrusive systems are discouraged because the tags might hamper the athletes' movement or, in event of body contact, even cause injuries. In some sports, intrusive techniques may violate sport regulations. Additionally, the update frequency of individuals' positions is limited due to the nature of radio signal and, due to time-slotting, decreases as the number of tracked individuals increases.

Video cameras used in computer-vision-based approaches, on the other hand, enable unobtrusive recovery of individuals' positions and trajectories. This can be done at comparatively high frequency, which is, especially if off-line processing is permissible, limited only by cameras' frame rate. However, computer-vision-based approaches have difficulties with maintaining individuals' identities over longer periods of time; many modern multi-view approaches rely on identification by tracking, meaning that they propagate the identities along the track, with little or no appearance-based validation. This can lead to propagation of identity switches when individuals come close and then disperse again. This problem is even more evident in uniformed environments, such as sports or high-security facilities, where individuals become visually indistinguishable due to similar clothing.

In this paper, we present a person detection and localization system that performs tracking by identification. The proposed system is composed of two subsystems. The first component is a commercially-available localization solution that is based on Ultra-Wideband radio technology. The second component is a state-of-the-art computer-vision based system that performs identification by tracking; anonymous detections are obtained from multiple views on frame-by-frame basis and then linked into trajectories by a global optimization method. The proposed combination performs all three steps—detection, localization and identification of individuals—on a frame-by-frame basis, and only then links these independent, identified, detections into tracks. Due to use of radio tags, the proposed system is currently suitable for closed-world surveillance applications and intelligent environments; however, we expect that eventual progress in miniaturization of radio tags will enable wider adoption in sports applications as well.

The subsystems are combined in two stages. In the first stage, we fuse information from the radio-based system directly into the camera-based detection algorithm to improve anonymous person detection and localization. In the second stage, we augment the anonymous detections with identity information from the radio system, before linking them into trajectories and thereby performing tracking by identification. We present evaluation methodology and metric, which is based on 2-D localization error, and use it to evaluate the performance of the system and its individual components. The experimental results on a challenging indoor dataset show that the proposed combination of computer vision and radio performs better, both in terms of localization error and maintaining the identities of individuals. To enable further research in the field of multi-modal sensor fusion, we offer our dataset to the research community as a free download [5]. To the best of our knowledge, this is the first publicly-available multi-modal dataset combining multiple calibrated cameras and a radio-based localization system.

The remainder of the paper is organized as follows. In Section 2, we provide an overview of related work in the field of person detection and tracking using multiple cameras, multi-modal sensor fusion and existing metrics for their performance evaluation. In Section 3 we introduce both components of our system, the radio-based and the computer-vision-based localization system. This is followed by both stages of fusion, presented in Section 4; the first stage improves the anonymous detection and localization, and the second stage results in tracking by identification. Section 5 describes the evaluation methodology and metric that we use to evaluate the proposed system and its components. Finally, in Section 6, we present experimental validation of the proposed approach, along with the dataset that we use, and conclude the paper in Section 7.

## 2. Related Work

The task of tracking multiple people using video cameras has a long tradition in the field of computer vision; an overview of existing multi-target tracking literature can be found in [2]. Here, we focus on approaches that use multiple cameras with overlapping fields of view, as such are usually required for recovery of individuals' positions and trajectories in the world coordinate system. The existing approaches can be roughly divided into two groups. The first are so-called detection-by-tracking approaches, which rely on sequential detection, usually using Kalman filter (e.g., [6,7]) or particle filters ([8,9]). Such trackers are causal; they consider only previously-processed frames, which is why they still represent state-of-the-art in the real-time tracking. However, relying on recursive detection may result in irrecoverable errors when a person fails to be detected or when detections are incorrectly linked. Such errors tend to propagate and multiply in the subsequent frames, eventually causing a tracker to fail. Tracking-by-detection approaches mitigate this issue by first employing robust frame-by-frame detection [10,11], on top of which global optimization methods are applied for tracking (e.g., [12]), usually off-line and in batch manner. However, when it comes to maintaining the identities of tracks, these approaches perform identification by tracking; they rely on identity propagation along the track, with none or limited appearance-based validation, and as such, they are prone to propagation of identity switches after people come close and disperse again. Recently, Shitrit *et al.* [13], extended state-of-the-art tracking approach [12] to preserve identities of players in sports, based on the color of their jerseys and their numbers. However, to reliably distinguish between individuals, a much larger assortment of visual cues is usually needed.

In their detection stage, many tracking-by-detection approaches aggregate information from multiple cameras using an occupancy map. Occupancy map, also referred to as occupancy grid or plan view, is a well-established concept in mobile robotics [14]. It involves discretizing the ground plane of area of interest into a grid, followed by estimation of occupancy probability for each of its cells. It was introduced in the field of computer vision with works of Beymer [15] and Yang *et al.* [16], both in the context of estimating the number of people in a room. In the work of Franco and Boyer [17], an occupancy map is used as a framework for fusion of multi-view silhouette cues. These approaches are bottom-up; they project points of the foreground likelihood, either stereo disparities of foreground regions obtained by background subtraction, of each view into the ground plane and then infer the occupancy likelihood from the amount of points projected in each cell. Works of Delannay *et al.* [18] and Muoz [19] also fall into this category. Similarly, Khan and Shah [11] warp the foreground regions from all views into a reference plane, producing a 2-D grid of occupancy likelihoods, which they call a synergy map. They use multiple planes, parallel to the reference plane, and stack the resulting synergy maps into a 3-D volume representing sampled scene space. Losada *et al.* [20] use multiple calibrated cameras and 3-D occupancy grid for localization of multiple mobile robots in an intelligent environment.

The state-of-the-art Probabilistic Occupancy Map (POM) by Fleuret *et al.* [10] uses a top-down approach. A generative model that approximates silhouettes with rectangles is used to back-project currently estimated occupancy probabilities into all views; the occupancy map is obtained by iterative optimization of the probability field, so that the difference between the back-projected and the input binary images, obtained by background subtraction, is minimized. Due to iterative nature and use of back-projection, the algorithm implicitly handles occlusions between people. Berclaz *et al.* [21] use the same framework, but instead of foreground images, output of a people detector is used. Alahi *et al.* [22] also obtain occupancy map from foreground images in a top-down manner, using sparsity-constrained inverse problem formulation.

The anonymous detections obtained by aforementioned approaches can be incorporated into a tracking framework, resulting in tracking by detection. In [11], graph cuts are used to link the frame-by-frame-detections, while in [10], tracking is done using dynamic programming and a local color appearance model. In recent work by Berclaz *et al.* [12], multi-object tracking on top of an occupancy map is formulated as a global optimization problem and solved using K-Shortest Paths algorithm. Their approach completely ignores the appearance and yet it has been shown to outperform other methods, therefore it represents state of the art in identification by tracking. Recently, Shitrit *et al.* [13] extended this approach to preserve identities based on sparsely-available visual cues. In our system, we achieve tracking by identification by augmenting anonymous detections with identity information from a radio-based localization system.

Fusion of visual information with other sensor modalities is also an active research topic. A popular choice appears to be a combination of one or more video cameras and a laser range finder, especially in mobile robot navigation (e.g., [23]), but also for pedestrian detection on autonomous vehicles and in driver assistance systems. For example, in [24], multiple baselines and a laser ranger finder are fused, whereas [25] proposes a combination of a stereo camera and a laser scanner. In [26], multiple sensor modalities—GPS, inertial sensors, stereo camera and a-priori 3-D model of environment—are used to improve pedestrian positioning accuracy in an urban environment. The work of Meingast *et al.* [27] proposes a method for automatic calibration of a heterogeneous sensor network, in which nodes consist of a video camera and a wireless sensor mote. This is achieved via fusion of data coming from both types of sensors; cameras are auto-calibrated up to scale factors from correspondences, obtained from detected moving objects, whereas scale factors are estimated with help of wireless motes and radio interferometry. A prominent area of multi-modal sensor fusion is also fusion of audio-visual information [28], which also deals with problem of localization and identification of speakers in a room. For this task, Bayesian networks with particle-filter-based inference are commonly used.

An overview of commonly used technologies and techniques for radio-based object detection and localization can be found in the survey paper by Liu *et al.* [3]. In addition to ubiquitous IEEE 802.11 (WiFi) wireless network infrastructure, Radio-Frequency Identification (RFID) and Ultra-Wideband (UWB) radio technology are commonly used. In case of passive RFID, the radio tag must pass close by the RFID reader to be detected. Such technology can be used for tracking assets and individuals in large-scale environments (*i.e*., whole building, campus), by equipping them with RFID tags and placing RFID readers at checkpoints. In [29], a combination of passive RFID and cameras is proposed to avoid exposing privacy of authorized people in video streams; if a person authenticates themselves with an RFID card when entering a room, their faces in video stream are blurred. Active RFID enables detection of more distant tags. Therefore, it can be also used for navigation in large-scale environments, by equipping a person or robot with RFID reader and using tags as landmarks [30]. RFID has also been fused with single camera with aim of improving object and human recognition [31,32].

WiFi and active RFID are commonly used for localization using Received Signal Strength Indication (RSSI), where distance between emitter and receivers is estimated from signal strength. This allows person and asset localization and tracking in large-scale environments, and also navigation using RSSI fingerprints of WiFi access points. There have been several attempts at fusing computer vision and WiFi signal strength for person localization, e.g., [33,34]. Anne *et al.* [35] propose indoor person localization by combining computer vision, WiFi signal strength and RFID, where RFID tags are installed in the floor and RFID reader is placed in individual's shoes. Cucchiara *et al.* [36] propose a combination of a network of video cameras and an active RFID system for detection of intruders in wide open areas. However, contrary to the system we propose, their system is not concerned with localization of individuals; their division of area of interest in relatively crude locations is done solely to allow association of detections from video cameras with those from the RFID system. Yu and Ganz [37] aim to prevent identity switches in longer video sequences by associating identities from a signal-strength-based active RFID system with tracklets that are obtained from a calibrated video camera. Later, they extended their approach to use raw (uncalibrated) radio measurements, and perform tracking and identity association in image plane instead on the ground plane [38].

Ultra-Wideband (UWB) radio [39], due to high bandwidth of signals, offers much higher temporal (and thus spatial) resolution than RFID-based solutions. Due to increased resolution, in addition to RSSI, Angle of Arrival (AoA), Time of Arrival (ToA) and Time Difference of Arrival (TDoA) can be used to precisely localize objects in smaller areas, such as a single room. Several commercially-available systems based on UWB are available on the market; in this paper, we use the solution offered by Ubisense [40], which localizes radio tags using AoA and TDoA measurements. Similarly to this paper and our previous work [41], Dibitonto *et al.* [42] also propose fusion of Ultra-Wideband radio localization system and computer vision. However, they use a single camera, and are concerned only with correlation of trajectories and not with localization performance.

When it comes to evaluation of localization results, different metrics are used by different authors. For example, in their survey paper on localization systems, Hightower *et al.* [1] suggest that researchers report 2-D localization error distribution. However, this information does not include information about missing and phantom detections. Several authors of computer-vision-based approaches ([10,18,21,22]) report number of false positive and negative detections or related metrics from information retrieval theory, such as precision and recall or false positive and false negative rate. Authors of [10,11] additionally report average localization error. Yet, although all perform localization on ground plane, some perform evaluation in image plane and some on ground plane, and they use different criteria to determine whether a detection is a true positive, a false positive or a false negative. Recent approaches [12,13] have adopted CLEAR Multi-Object Tracking (MOT) metrics [43]. However, it appears that the preferred formulation is the one from [44], which is based on evaluation in the image plane using bounding box overlap. While suitable for monocular approaches that perform detection and tracking in the image plane, such evaluation is less suitable for approaches that perform detection and tracking in world coordinate system, where, as argued in Section 5, localization error on ground plane should be considered instead.

## 3. Radio-Based System and Computer-Vision-Based System

In this section, we present the radio-based system and the computer-vision-based system, which are the main components of our tracking-by-identification system. Table 1 summarizes the comparison of radio-based and camera-based detection and localization, which is based on our previous work [41] and re-confirmed by experimental results in Section 6.2. As can be seen, the radio-based system provides very reliable detection, with practically no false positive and negative detections, but with considerable localization error in a cluttered environment. On the other hand, computer-vision-based approach offers better localization, but the detection sometimes suffers from false positives and false negatives. These findings form the baseline for fusion in Section 4.

### 3.1. Radio-Based System

**Figure 1.**
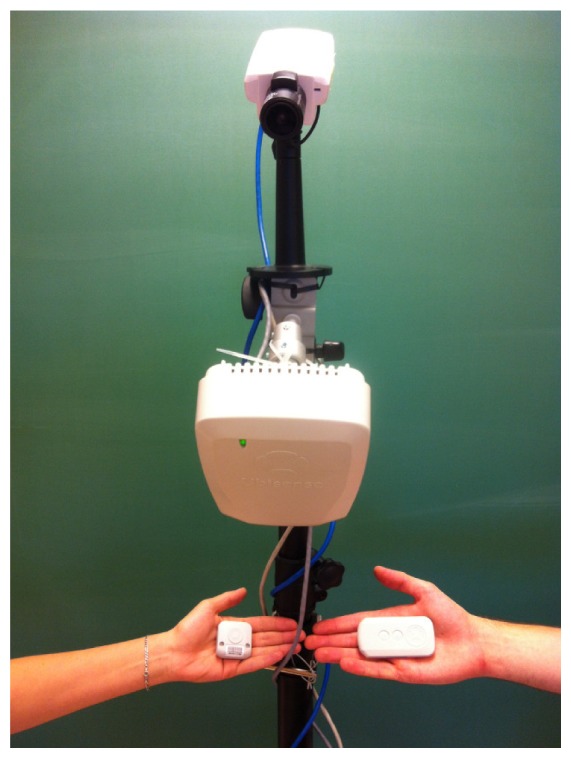
An AXIS P1346 IP camera and a Ubisense sensor mounted on a stand. The hand on the left side is holding a Ubisense compact tag, while the hand on the right side is holding a slim tag.

The radio-based localization system that we use is a commercially-available solution from Ubisense [40]. It is based on Ultra-Wideband (UWB) radio technology [39] and comprises a network of radio receivers (sensors) that are installed in a room and small radio-emitting tags that are worn by people. There are two types of tags available—omnidirectional compact tags (http://www.ubisense.net/en/resources/factsheets/series-7000-compact-tag.html) and directional slim tags (http://www.ubisense.net/en/resources/factsheets/series-7000-slim-tag.html), both of which are shown in Figure 1. Tags are localized with combination of Angle-of-Arrival (AoA) and Time-Difference-of-Arrival (TDoA) measurements [3], which makes 3-D localization possible even when only two sensors detect a tag.

To prevent interference between tags, each tag is allocated time slots during which it emits signal. Combined with limitations stemming from the nature of the radio signal, this caps the position refresh frequency for a tag at 33.75 Hz, which is achievable when only a single tag is used and decreases with increasing number of tags. Using five tags, we measured the median update frequency of an individual tag to be 4.6 Hz.

For each tag, its position and unique identifier can be obtained from system's software platform. Since identity is encoded in the radio signal, the system inherently does not suffer from identity switches, and can be considered as a tracking-by-identification system. The advertised localization accuracy of the system is 15 cm, with 99% of errors being within 30 cm, however our experiments indicate that performance in a realistic, cluttered indoor environment is much lower due to occlusions (both from other people and from inanimate objects) and presence of metallic surfaces, which reflect radio signals. These cause non-line-of-sight (NLOS) signal propagation, which is the main cause of errors in radio-based localization.

### 3.2. Computer-Vision-Based System

The computer-vision-based system uses multiple calibrated and time-synchronized video cameras with overlapping fields of view. It is based on the state-of-the-art Probabilistic Occupancy Map (POM) algorithm [10] for frame-by-frame person detection and localization, and K-Shortest Paths (KSP) [12] algorithm for linking independent detections into trajectories.

The POM algorithm operates by discretizing the ground plane of area of interest into a rectangular grid, typically of resolution 20 cm. It then, on frame by frame basis, iteratively estimates the probabilities of occupancy for all cells in the grid, based on input binary images, which are usually obtained by means of background subtraction. It uses a simple and robust appearance model that approximates the silhouette of a person at each possible location with a rectangle of fixed height. Typically, rectangles correspond to width of 50 cm and height of 175 cm. Using this model, currently estimated occupancy probabilities are back-projected into input views. Estimation begins by assigning all cells uniform initial probabilities of occupancy, and then the probability field (occupancy map) is iteratively optimized so that the difference between input binary images and synthetic back-projected images is minimized.

Here, we briefly summarize the mathematical model used by POM, as we refer to it in Section 4.1; for complete formulation, see [10]. Given a discretization of ground plane in *N* cells, we denote by **X** a vector of binary random variables (*X*_1_,…, *X_N_*), standing for the occupancy of each cell (*X_k_* = 1 if *k*-th cell is occupied and *X_k_* = 0 if it is vacant). The goal is to estimate the posterior probability over occupancy maps, *P*(**X**∣**B**), given the information from *C* video cameras **B** = (*B*_1_,…,*B_C_*). This problem is, under independence assumption, broken down into estimating probability of occupancy for each cell, *P*(*X_k_* = 1∣**B**). The authors derive expression for estimated probability that *k*-th cell is occupied, *q_k_* = *Q*(*X_k_* = 1), via minimization of Kullback-Liebler divergence between the estimated posterior *Q*(**X**∣**B**) and the “true” posterior *P*(**X**∣**B**). Here, we write the expression from [10] in a bit more general form:

(1) $$q_{k}=\frac{1}{1+\exp\{\lambda_{k}+\underset{c}{\text{∑}}O_{c}(k,X)\}}$$

where *λ_k_* is log-ratio of the prior probabilities,

(2) $$\lambda_{k}=\text{log}\frac{P(X_{k}=0)}{P(X_{k}=1)}$$

and 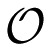
*_c_*(*k*, **X**) is a term that encapsulates all information from the *c*-th camera about the occupancy of *k*-th cell:

(3) $$O_{c}(k,X)=E_{Q}\{\log P(B_{c}|X)|X_{k}=0\}-E_{Q}\{\log P(B_{c}|X)|X_{k}=1\}$$

In the above equation, *E_Q_*{·} denotes the expectation under the approximation **X** ∼ *Q*. In the original formulation, *B_c_* denotes binary image obtained from view *c* using background subtraction, however Equation (3) is general enough to allow inclusion of any sensor modality

For visual sensors (cameras), a generative model that relates the values of **X** to the input binary images is used; at each iteration step, for each view, it generates a synthetic image from the currently estimated values of **X**. The authors define a normalized pseudo-distance between the input binary image *B_c_* and a synthetic image *A_c_*, which roughly corresponds to pixel overlap between the two. The conditional distribution is then modeled as $P(B_{c}|X)=\frac{1}{Z}e^{-\Psi (B_{c},A_{c})}$ where Ψ(*B_c_*,*A_c_*) denotes the pseudo-distance between the images. In order to make the problem solvable, the following approximation is made under assumptions that are detailed in [10]:

(4) $$E_{Q}\{\Psi (B_{c},A_{c})|X_{k}=\xi \}\approx \Psi (B_{c},E_{Q}(A_{c}|X_{k}=\xi ))$$

The term in Equation (3) therefore becomes:

(5) $$O_{c}(k,X)=\log\frac{e^{-\Psi (B_{c},E_{Q}\{A_{c}|X_{k}=0\})}}{e^{-\Psi (B_{c},E_{Q}\{A_{c}|X_{k}=1\})}}=\Psi (B_{c},E_{Q}\{A_{c}|X_{k}=1\})-\Psi (B_{c},E_{Q}\{A_{c}|X_{k}=0\})$$

The conditional synthetic images *E_Q_*(*A_c_*|*X_k_* = *ξ*), *ξ* = {0, 1} correspond to the average synthetic images *E_Q_*{*A_c_*} with *q_k_* forced to 0 and 1, respectively. The first case represents the hypothesis that the *k*-th cell is vacant, and the second that it occupied.

For each frame, a set of anonymous detections is obtained in the form of cells with high resulting probability of occupancy. These are linked into trajectories using K-Shortest Paths (KSP) tracker [12], which formulates the tracking task as a global optimization problem on a Directed Acyclic Graph, resulting in a convex optimization function. The obtained trajectories inherently have no identities; these must be assigned manually, for example using the identity of a person that the trajectory was initialized on. This is state-of-the-art identification-by-tracking, since linking is based solely on spatio-temporal proximity. Linking is performed off-line and in batch manner.

To capture video, we use Axis P1346 IP cameras (http://www.axis.com/products/cam_pl346) with wide-angle lenses. Therefore, lens distortion is first calibrated and corrected using OCamCalib toolbox [45] and then intrinsic and extrinsic parameters are calibrated on rectified images using Camera calibration toolbox for Matlab [46]. The binary foreground images are obtained using implementation of background subtraction algorithm [47,48] provided by OpenCV library. We use publicly-available implementations of POM (http://cvlab.epfl.ch/software/pom) and KSP algorithm (http://cvlab.epfl.ch/software/ksp).

For computer-vision-based system, the data capture frequency is limited only by cameras' frame-rates, in our case 20 frames per second. This is also the highest possible temporal resolution of the position data, which is achievable by offline processing. Since the detection algorithm relies on back-projection, its speed depends both on the number of locations (occupancy map resolution) and the size of input images. For illustration, times required to process a single four-view frame with grid resolutions 25 cm (899 locations) and 10 cm (5,396 locations) are listed in Table 2. Since the algorithm uses very simple appearance model, there is no significant change in result as images are scaled down in order to gain processing speed. The tracking step is applied in batch mode, therefore all data processing is assumed to be performed off-line, with position update frequency of 20 Hz.

## 4. Fusion of Systems

In this section, we present the fusion of both systems that were introduced in Section 3. The most obvious advantage of the radio-based system is that it provides detections with reliable identity information, even though their localization might be relatively poor. On the other hand, anonymous camera-based detections are generally better localized, although they occasionally suffer from gross errors. Therefore, the logical way to fuse both systems is to combine the best of both worlds: good camera-based localization and reliable radio-based identities. This is achieved in two stages. In the first stage, described in Section 4.1, we improve anonymous detection and localization of camera-based algorithm by adding location information from the radio system. The resulting anonymous detections are then augmented with identity information from radio tags in the second stage, which is described in Section 4.2, and results in tracking by identification.

### 4.1. Improvement of Anonymous Detection and Localization

The first fusion stage aims to improve anonymous detection and localization. We use the approach from our prior work [41] and expand it by offering a different, more formal view on how it operates. The idea of the approach is to anonymize detections from radio-based system and fuse them directly into the POM algorithm in order to reduce the number of gross errors (false positive and false negative detections) it produces. This is done by introducing additional data term, 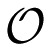
*_radio_*(*k*,**X**), in Equation (1):

(6) $$q_{k}=\frac{1}{1+\exp\{\lambda_{k}+O_{\text{radio}}(k,X)+\underset{c}{\text{∑}}O_{c}(k,X)\}}$$

The term 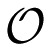
*_radio_*(*k*,**X**) encapsulates all radio system's information about *k*-th cell's occupancy. Generally, it is of form for sensor data terms from Equation (3), however, since we model it as being independent of currently estimated probabilities **X**, it is simplified to:

(7) $$O_{\text{radio}}(k,X)=O_{\text{radio}}(k)=\log\frac{P(R|X_{k}=0)}{P(R|X_{k}=1)}=-\log\omega_{k}$$

where *R* denotes information from the radio system. As per [41], we model the inverse of the likelihood ratio in the above equation as:

(8) $$\omega_{k}=\frac{P(R|X_{k}=1)}{P(R|X_{k}=0)}=\alpha \cdot \underset{t}{\max}\{G_{t}(x_{k},y_{y})\}+\beta$$

where *G_t_* is a Gaussian centered on the coordinates of tag *t* detection, (*x_t_*,*y_t_*), and evaluated at the center of the cell *k*, (*x_k_*,*y_k_*):

(9) $$G_{t}(x_{k},y_{k})=e^{-\frac{{\left(x_{k}-x_{t}\right)}^{2}+{\left(y_{k}-y_{t}\right)}^{2}}{2\sigma_{t}^{2}}}$$

We can see from Equation (6) that, since they are both independent from currently estimated probablities **X**, we can combine terms *λ_k_* and 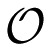
*_radio_*(*k*) into a new log prior ratio term:

(10) $$\lambda_{k}^{\prime }=\lambda_{k}\cdot O_{\text{radio}}(k)=\log\frac{P(X_{k}=0)}{P(X_{k}=1)}\cdot (-\log\omega_{k})=\log\frac{P(X_{k}=0)}{\omega_{k}\cdot P(X_{k}=1)}$$

Let *ρ_k_* denote the old prior, *ρ_k_* = *P*(*X_k_* = 1), and $\rho_{k}^{\prime }$ the new prior, $\rho_{k}^{\prime }=P^{\prime }(X_{k}=1)$:

(11) $$\lambda_{k}^{\prime }=\log\frac{1-\rho_{k}^{\prime }}{\rho_{k}^{\prime }}=\log\frac{1-\rho_{k}}{\omega_{k}\cdot \rho_{k}}$$

From Equation (11), we can derive the expression for the new occupancy prior for *k*-th cell:

(12) $$\rho_{k}^{\prime }=\frac{\omega_{k}\cdot \rho_{k}}{1-\rho_{k}(1-\omega_{k})}$$

**Figure 2.**
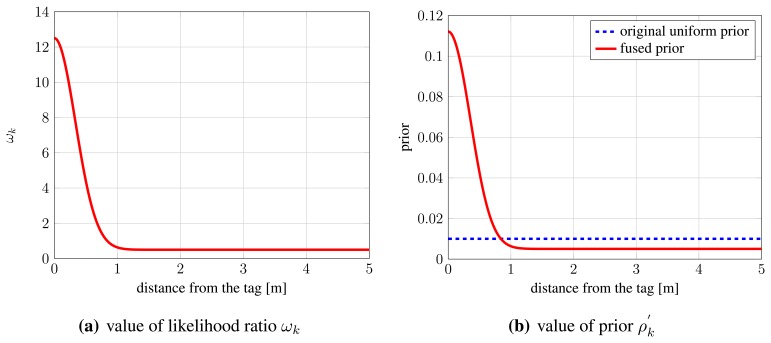
The value of likelihood ratio *ω_k_* and resulting fused prior.

Therefore, we can see the proposed fusion of information from radio-based system as introduction of non-uniform priors, with high values in cells close to locations where radio tags are detected and low values in cells far away from such locations. Figure 2(a) shows the value of likelihood ratio *ω_k_* from Equation (8) as the distance from a radio tag increases. We use the parameters from [41]: *α* = 12, *β* = 0.5 and default uniform prior for POM, *ρ_k_* = 0.01. From the results presented in Section 6.2, we have estimated *σ_t_* = 1/3 m. Figure 2(b) shows the resulting prior value $\rho_{k}^{\prime }$ from Equation (12). As can be seen, the chosen parameters result in prior that is, compared with the static uniform prior, roughly eleven times higher in cells that contain radio system tags, and roughly halved for cells that are far away from cells containing radio tags.

Consequently, given the same visual evidence, a cell will more likely be recognized as occupied when a radio tag is present in its vicinity In such fusion scheme, the majority of information required for detection is provided by the cameras, whereas information from radio system increases robustness. In cases when the ambiguity of visual information causes the algorithm to favor convergence to an incorrect solution, yet there is also sufficient visual evidence for the correct solution, the modified prior helps the algorithm to converge to the correct solution. On the other hand, even if information from radio system is incorrect, it does not disrupt the algorithm's convergence to the correct solution, as long as visual information is good.

### 4.2. Tracking by Identification

In the second stage, we augment the obtained anonymous detections with identity information from radio tags. As shown in Section 3, radio tags offer reliable identity information, while their localization might be relatively poor. Conversely, computer-vision-based system produces well-localized anonymous detections, with some gross errors corrected by the first stage of fusion, described in Section 4.1.

The augmentation of anonymous detections with identity information is done on frame-by-frame basis. At each time instant, the first stage of fusion results in *P* anonymous detections, comprising their coordinates on ground plane: $d_{i}^{\text{anon}}=(x_{i}^{\text{anon}},y_{i}^{\text{anon}})$; *i* = 1…*P*. Similarly, radio system produces *R* identified detections, consisting of coordinates on ground plane and identity information, denoted by *γ*: $d_{j}^{\text{radio}}=(x_{j}^{\text{radio}},y_{j}^{\text{radio}},\gamma_{j}^{\text{radio}})$; *j* = 1…*R*. We find optimal assignment between both sets of detections based on their spatial configuration. We construct a cost matrix *M* with costs of assigning each detection $d_{i}^{\text{anon}}$ to each detection $d_{j}^{\text{radio}}$, based on Euclidean distance between them, and find optimal assignment using Hungarian method [49]:

(13) $$\begin{array}{cc} M & \begin{array}{ccc} d_{1}^{\text{anon}} & \cdots & d_{P}^{\text{anon}} \end{array}\\ \begin{array}{c} d_{1}^{\text{radio}}\\ \vdots \\ d_{R}^{\text{radio}} \end{array} & \begin{array}{ccc} m_{11} & \cdots & m_{1P}\\ \vdots & \ddots & \vdots \\ m_{R1} & \cdots & m_{RP} \end{array} \end{array}$$

(14) $$m_{ij}=\left\|d_{i}^{\text{anon}}-d_{j}^{\text{radio}}\right\|=\sqrt{{\left(x_{i}^{\text{anon}}-x_{j}^{\text{radio}}\right)}^{2}+{\left(y_{i}^{\text{anon}}-y_{j}^{\text{radio}}\right)}^{2}}$$

This assumes that although individual radio tags might be poorly localized, their overall spatial configuration remains similar to that of the anonymous detections, which turns out to be a reasonable assumption most of the time. We obtain new identified detections in following way:

- For each assigned pair $\left(d_{j}^{\text{radio}},d_{i}^{\text{anon}}\right)$, we augment coordinates of $d_{i}^{\text{anon}}$ with identity from $d_{j}^{\text{radio}}$.
- For all unassigned $d_{j}^{\text{radio}}$ (happens when *R* > *P*), their original coordinates are used.
- All unassigned $d_{i}^{\text{anon}}$ (happens when *R* < *P*) are discarded as false positives, as our system assumes that all individuals wear radio tags. In a different application, for example an intruder detection system, this information could be used differently.

The above procedure results in *R* new identified detections:

(15) $$d_{j}^{\text{result}}=\{\begin{array}{ll} \left(x_{i}^{\text{anon}},y_{i}^{\text{anon}},\gamma_{j}^{\text{radio}}\right) & \text{if}\;d_{j}^{\text{radio}}\;\text{is assigned};(d_{j}^{\text{radio}},d_{i}^{\text{anon}})\\ \left(x_{j}^{\text{radio}},y_{j}^{\text{radio}},\gamma_{j}^{\text{radio}}\right) & \text{otherwise} \end{array}$$

(16) j=1⋯R

These frame-by-frame identified detections can either be used on their own, or additionally post-processed by applying a tracking step to smooth the resulting trajectories and correct possible gross errors, as we illustrate in Section 6.4. In this case, each sequence of identified detections is separately linked into an identified trajectory, thereby performing tracking by identification.

## 5. Evaluation Methodology

In this section, we present our evaluation methodology, which is based on 2-D localization error on ground plane. We believe this is appropriate for evaluating person detection and tracking systems that perform localization in the world coordinate system, even for approaches that are based solely on computer vision. Evaluation with bounding box overlap, as suggested by [44], is suitable only for monocular approaches that perform detection and tracking in the image plane. It is less suitable for multi-view approaches that perform detection and tracking in world coordinate system, where such an evaluation would favor approaches whose appearance model (*i.e*., the size of bounding boxes) matches that of the ground-truth annotations. This might not be fair, as due to multi-view geometry, better-fitting bounding boxes in individual image planes might not necessarily mean better localization on the ground plane. Furthermore, it is specific to computer-vision-based approaches and does not allow evaluation of approaches based on other sensor modalities. It is worth noting that MOT metrics, based on 2-D localization error on ground plane, have been used in certain tasks of CLEAR evaluation, such as 3-D visual person tracking [43].

Our evaluation methodology is based on the same idea as the general formulation of the MOT metrics. At each time instant, we wish to find mapping between given detections and ground-truth annotations, and then estimate both the localization error and the amount of missing and false positive detections. However, in contrast to Multiple-Object Tracking Accuracy (MOTA) metric, which merges statistics for all error types into a single number, we report statistics for each error type separately. We believe this both offers better insight into system's performance and also allows easier comparison; it should be up to the person performing the comparison to compute the single-number metric from all the components and weight them according to the requirements of specific application. Furthermore, we find the mapping between detections and ground-truth annotations using different cost functions, each resulting in a mapping that reveals a different aspect of a system:

- *detection*: does the system correctly determine the number of individuals in a room (*i.e*., are there any false positive and missing detections)
- *localization*: how well does the system determine positions of detected individuals
- *identification*: how well does the system determine/maintain identities of detected individuals (if it performs identification at all)

As shown in the following subsections, the three aspects are not always completely de-coupled. For example, a co-occurring false positive and false negative detection cannot be distinguished from a detection with a gross localization error. Similarly, identity switches also manifest themselves as gross localization errors.

### 5.1. Evaluation for Systems that Perform Anonymous Detection and Localization

A system that performs anonymous detection and localization is concerned only with recovering correct number of individuals in the room, along with their positions. Such system can be used in applications where positions of individuals are important, but their identities are not, for example in person counting or determining which locations are most frequented.

At each time instant (e.g., a video frame), the system produces *D* anonymous detection hypotheses, comprising their 2-D coordinates on the ground plane: $d_{i}=(x_{i}^{d},y_{i}^{d})$; *i* = 1…*D*. Similarly, we have a group of *G* anonymous ground-truth annotations on the ground plane: $g_{i}=(x_{j}^{g},y_{j}^{g})$; *j* = 1…*G*. Since we do not have any identity information that could be used for assigning detections to ground-truth points, we find an optimal assignment based on their spatial configuration. We construct a cost matrix *C*, whose elements describe the cost of assigning each detection d*_i_* to each ground-truth point g*_j_*:

(17) $$\begin{array}{cc} C & \begin{array}{ccc} d_{1} & \cdots & d_{D} \end{array}\\ \begin{array}{c} g_{1}\\ \vdots \\ g_{G} \end{array} & \begin{array}{ccc} c_{11} & \cdots & c_{1D}\\ \vdots & \ddots & \vdots \\ c_{G1} & \cdots & c_{GD} \end{array} \end{array}$$

Different cost functions can be used to obtain assignments that reveal different aspects of the system. In this paper, we define three different cost functions, and refer to their use for evaluation as using *Metric A*, *Metric B* and *Metric C*. The first two are presented in this subsection, whereas the third one is presented in the next one. Primarily, all three cost functions are based on the Euclidean distance between a detection and a ground-truth point:

(18) $$c_{ij}^{A}=c^{A}(d_{i},g_{j})=\left\|d_{i}-g_{j}\right\|=\sqrt{{\left(x_{i}^{d}-x_{j}^{g}\right)}^{2}+{\left(y_{i}^{d}-y_{j}^{g}\right)}^{2}}$$

**Figure 3.**
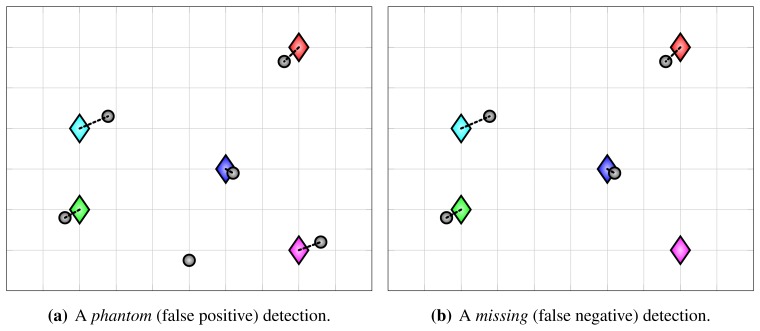
Examples of assignments obtained with proposed evaluation methodology, illustrating a false positive (**a**), and a false negative detection (**b**). Diamonds represent ground-truth points and gray circles represent anonymous detections. The dotted black lines denote assigned pairs.

The optimal assignment, which minimizes the overall assignment cost, can be found using Kuhn-Munkres algorithm (Hungarian method) [49]. If matrix *C* is non-square (*i.e., D* ≠ *G*), it should be augmented to square matrix by introducing dummy elements, which is often already handled by the algorithm implementations. After running the algorithm and obtaining the optimal assignment, the unassigned detections are considered to be false positive (phantom) detections and the unassigned ground-truth points are considered to be missing (false negative) detections. For assigned detection/ground-truth pairs, their distances *c_ij_* correspond to the resulting localization error. From now on, we refer to use of cost function *c^A^* from Equation (18) for constructing assignments as *Metric A*. Examples of assignments with a false positive and a false negative detection are shown in Figure 3.

**Figure 4.**
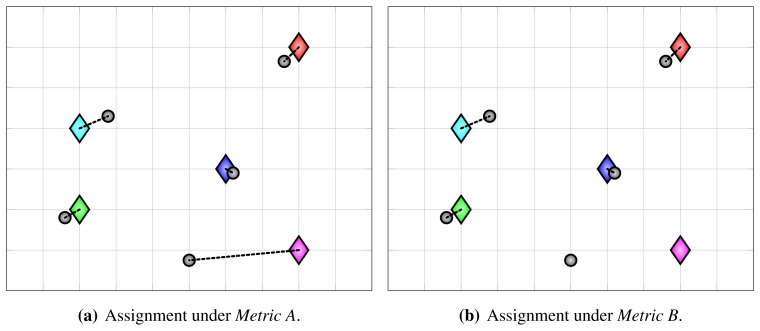
An illustration of co-occurring false positive and false negative detection. Since detections are anonymous, such detection errors are indistinguishable from gross localization errors. Evaluation using different cost functions for constructing assignments reflects this error in different ways. Under *Metric A*, the error manifests itself as a large localization error, as shown in (**a**). Under *Metric B*, the error manifests itself as a pair of false positive and false negative detections, as shown in (**b**).

**Figure 5.**
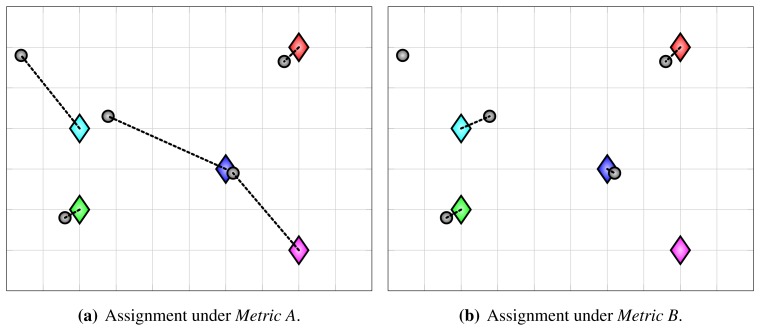
An illustration of a situation when co-occurring false positive and false negative detection affect whole assignment under *Metric A* (**a**). In such cases, *Metric B* results in more intuitive assignment, shown in (**b**).

Detection and localization errors cannot always be clearly distinguished. When using the cost function *c^A^* from Equation (18), a co-occurrence of a false positive and a missing detection manifests itself as a large localization error (Figure 4(a)), sometimes even affecting the whole assignment (Figure 5(a)). To mitigate this issue, we can modify the cost function to include gating. Whenever the distance between a detection and a ground-truth point exceeds a certain threshold, the cost is set to ∞, which means that the assignment algorithm is prevented from assigning that pair:

(19) $$c_{ij}^{B}=c^{B}(d_{i},g_{j})=\{\begin{array}{ll} \left\|d_{i}-g_{j}\right\| & \text{if}\left\|d_{i}-g_{j}\right\|\leq T_{d}\\ \infty & \text{otherwise} \end{array}$$

Modified assignments, obtained using the above cost function, which we refer to as *Metric B*, are shown in Figure 4(b) and Figure 5(b). Under *Metric B*, gross localization errors contribute to false positive and false negative statistics instead of localization error statistics.

The result of evaluation using either *Metric A* or *Metric B* includes the false positive and false negative statistics, and the localization error statistics. The latter can be given either in the form of mean error and standard deviation, or in the form of (cumulative) distribution plot. If ground-truth points have identities, the false negative statistics can be computed on per-person basis, whereas for false positives, only overall statistics can be provided, since we cannot tell to which person a phantom detection belongs. It should be noted that differences in update frequencies between systems can also be accounted for via localization error, if evaluation is performed at the frequency of the fastest system and positions from slower systems are held until new ones are obtained.

### 5.2. Evaluation for Systems that Perform Detection, Localization and Identification

In addition to coordinates on ground plane, systems that perform detection, localization and identification, either in form of tracking or on frame-by-frame basis, also include identity information *γ* in their detection hypotheses: $d_{i}=(x_{i}^{d},y_{i}^{d},\gamma_{i}^{d})$; *i* = 1…*D*. For evaluation, identity information must be supplied by ground-truth annotations as well: $g_{j}=(x_{j}^{g},y_{j}^{g},\gamma_{j}^{g})$. One way to obtain the mapping is to directly assign closest detections and ground-truth points with same identities, which is formally the same as using the following cost function, which we refer to as *Metric C*:

(20) $$c_{ij}^{C}=c^{C}(d_{i},g_{j})=\{\begin{array}{ll} \left\|d_{i}-g_{j}\right\| & \text{if}\;\gamma_{i}^{d}=\gamma_{j}^{g}\\ \infty & \text{otherwise} \end{array}$$

**Figure 6.**
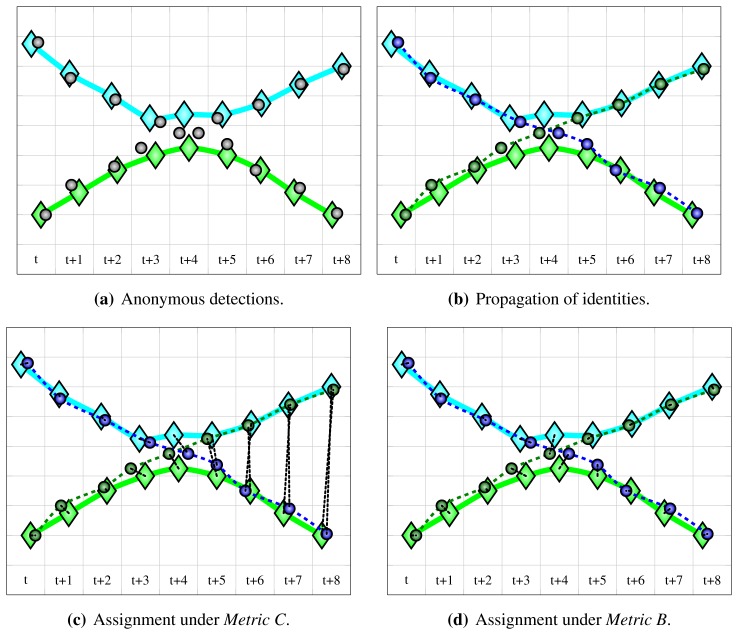
An illustration of a propagated identity switch occurring during identification by tracking. (**a**) shows ground-truth trajectories of two individuals (diamonds) and resulting anonymous detections (gray circles). When individuals come close together, localization becomes difficult. (**b**) shows identification by tracking, *i.e*., propagation of identities along tracks. Due to poor localization results, identity switch occurs at time instant *t* + 4 and is propagated further on. (**c**) and (**d**) show evaluation under *Metric C* and *Metric B* (cost functions *c^C^* and *c^B^* from Equations (20) and (19)), respectively. Resulting assignments are denoted by black dotted lines. Under *Metric C*, a propagated identity switch directly manifests itself as localization error. Under *Metric B*, identities of assigned pairs are compared after spatially-optimal assignment is obtained, and confusion matrix is constructed for detailed analysis.

In the resulting assignment, spurious multiple detections with same identity contribute to false positive detection counter, and missing detections to missing detection counter; since identity information is available both for detections and ground-truth points, per-person statistics can be obtained. Propagated identity switches manifest themselves as large localization error, as shown in Figure 6(c).

Even more detailed evaluation is possible using *Metric B* (cost function *c^B^* from Equation (19)), under which each detection is assigned to its closest ground-truth point, regardless of their identities (Figure 6(d)). This way, the resulting localization error reflects only system's (anonymous) localization performance. Then, identities of assigned detections and ground-truth points are compared, resulting in a confusion matrix—for an example, see Section 6.4. Unassigned detections contribute to “phantom detections” row and unassigned ground-truth points contribute to “missing detections” column. The rows of confusion matrix sum up to the number of all ground-truth points with corresponding identities, whereas columns sum up to the number of all detections with corresponding identities. Therefore, per-person precision and recall can be obtained by taking diagonal value and dividing it by row or column sum, respectively. The non-diagonal elements show how often a system mistook a given person for someone else.

Note that confusion matrix also allows computation of MOTA metric components. The ratio of misses can be computed as the sum of missing detections column over number of all detections (sum of all rows of confusion matrix). Similarly, ratio of false positives can be computed as the sum of phantom detections row over number of all detections. The ratio of instantaneous identity mismatches cannot be computed, as the proposed methodology has no notion of tracking; however, the ratio of global identity mismatches, proposed to be used instead by [13], can be computed as the sum of all non-diagonal elements over number of all detections.

## 6. Experimental Results

We validate the proposed tracking-by-identification system on a challenging indoor dataset, which we describe in Section 6.1. First, in Section 6.2, we separately evaluate detection and localization performance of each subsystem. Results of this evaluation have been used for summarized comparison in Table 1, which also served as the baseline for the fusion of both subsystems. This is followed by results for fusing both subsystems with the aim of improving anonymous detection and localization in Section 6.3, while the whole proposed tracking-by-identification system is evaluated in Section 6.4.

### 6.1. Dataset

To the best of our knowledge, there is no publicly-available dataset comprising video streams from multiple calibrated video cameras and data stream from a radio-based localization system. Therefore we captured and annotated a challenging dataset ourselves. As this is the first dataset of its kind, we make it publicly-available for other researchers to use [5].

**Figure 7.**
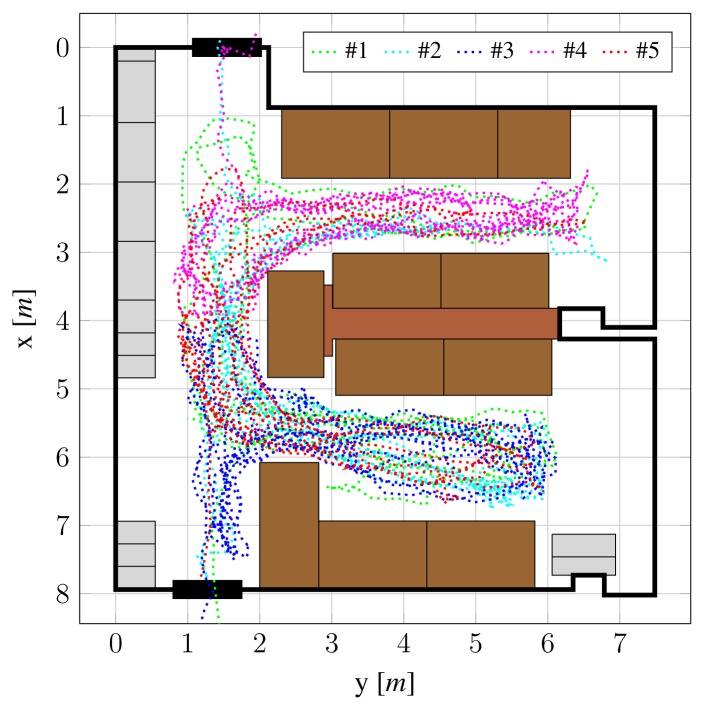
Top view of the room with ground-truth trajectories of the five individuals.

We captured the dataset by placing four Ubisense sensors and four Axis P1346 IP cameras into a 8.0 × 7.5 m room (Figure 7), at height 2.2 m.

**Figure 8.**
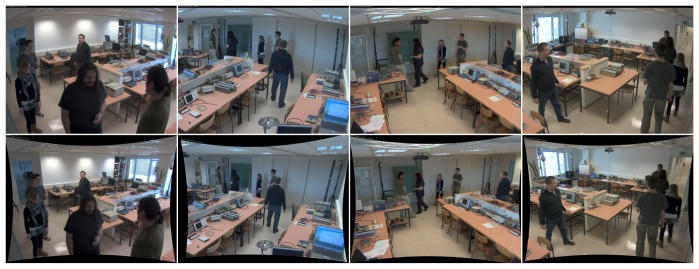
The views from all four cameras. Original images are shown in top row. We use images with lens distortion calibrated and corrected, as shown in the bottom row.

The views from cameras are shown in Figure 8. The room represents a realistically cluttered indoor environment, which is challenging both for radio-based and camera-based person localization. On one hand, difficulties in accurate and reliable camera-based position estimation arise due to occlusions of individuals, both among themselves and by inanimate objects, such as office furniture. On the other hand, the presence of radio-reflective metallic surfaces, in conjunction with obstacles, leads to multipath-related problems in radio-based localization.

Cameras and radio localization system were time-synchronized using a Network Time Protocol (NTP) server. Video from all four cameras was streamed at resolution 2,048 × 1,536 and 20 frames per second, and stored to hard drive using H.264 video codec. As indicated in Section 3.2, we downsampled the video streams to 512 × 384 before using them with POM for performance reasons. However, capturing the dataset in high definition broadens the possibilities for its further use.

Location events from the radio-based localization system were also stored in a file together with timestamps of their occurrence. The videos were later manually synchronized using timestamps printed on top of the frames, which are also used to associate radio tag location events with individual frames. Ground truth was annotated manually, by clicking on individuals' heads in each view and, using calibration information, reconstructing their 3-D coordinates.

The scenario of the dataset involves five people, equipped with radio tags, walking around the room. At frame #231, individuals begin to enter the room, and by frame #551, all five of them are inside. After walking around for roughly two minutes, they begin to leave at frame #2861. By frame #3211, everyone has left the room and additional lights have been turned on, changing the illumination. Individuals begin to re-enter the room at frame #3481, and by #3721 everyone is inside again. After walking around for 200 seconds, people begin to remove their radio tags at frame #7761 and then proceed to leave the room.

Since the radio signal penetrates through walls, the radio-based system tends to detect tags even before they enter the room, and localization error makes them appear inside the room. During evaluation, this would result in false positive detections. Therefore, to make the comparison fair, we evaluate only on the portions of dataset where all people are present in the room. Specifically, we split the dataset into two non-overlapping parts. The first part encompasses frames 551–2861, which amounts to approximately two minutes, and is used to evaluate detection and localization performance of subsystems. The second part, consisting of frames 3721–7761 (200 s), is used to evaluate both stages of proposed fusion.

### 6.2. Evaluation of Subsystems' Detection and Localization

To gain insight into strengths and weaknesses of both subsystems, we separately evaluate each of them. At this point, we are interested only in their detection and localization performance. From their nature, we already know that radio tags carry the identity information, which, in itself, is reliable, whereas camera-based detections provide no identity information unless it is manually assigned to the trajectories produced by the tracker. Evaluation and comparison are performed using methodology from Section 5.1, on the first part of the annotated dataset (frames 551–2861).

**Figure 9.**
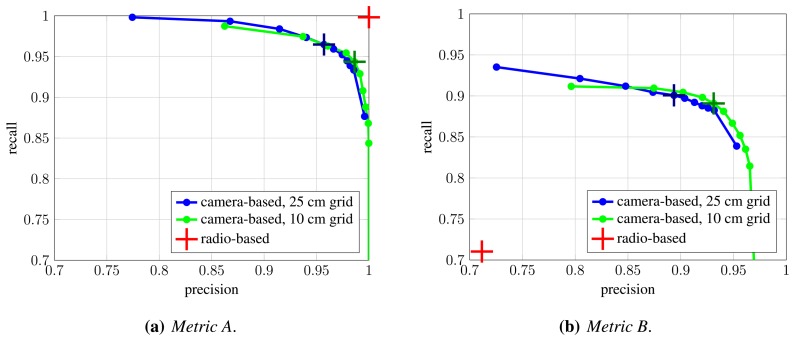
Comparison of camera-based and radio-based detection and localization: precision and recall curves under *Metric A* (**a**) and *Metric B* (**b**). The colored curves represent precision-recall curves for two occupancy map resolutions as *σ_pom_* parameter is varied. The crosses on the curves denote the operating point we chose for further experiments, *σ_pom_* = 0.005. The red cross denotes the result of radio-based localization system.

We evaluate two occupancy map resolutions: 25 cm, which is used by authors of [10], and 10 cm. In both cases, silhouettes are approximated with rectangles that assume a person's width and height to be 50 cm and 175 cm, respectively. Figure 9 shows obtained precision and recall values when assignments are constructed under *Metric A* and *Metric B*. For the latter, we chose the distance threshold that corresponds to the assumed width of a person, *T_d_* = 0.5 m. The curves for camera-based detection are obtained by varying the *σ_pom_* parameter, which controls the desired fitting between the input binary images and back-projected synthetic images, and therefore influences the compromise between resulting occupancy map's precision and recall. Table 3 shows precision, recall and mean localization error for several operating points (*σ_pom_* values) of camera-based system and a single operating point of radio-based system.

Under *Metric A*, which evaluates detection performance, the radio-based system achieves perfect precision and recall (red cross in Figure 9(a)), since there are practically no false positive and negative detections. However, as indicated by evaluation under *Metric B*, significant portion of these detections are poorly localized, which is reflected in the reduced precision and recall under this metric (red cross in Figure 9(b)). Conversely, it can be seen that false positive and false negative detections are more common in computer-vision-based approach, but at the same time, obtained detections are much better localized. The difference between Figure 9(a) and 9(b) is significantly smaller for computer-vision-based system, while quite obvious for the radio-based one. As expected, occupancy map with higher resolution results in better detection and localization. Based on the results, we chose *σ_pom_* = 0.005 as the operating point for further experiments.

**Figure 10.**
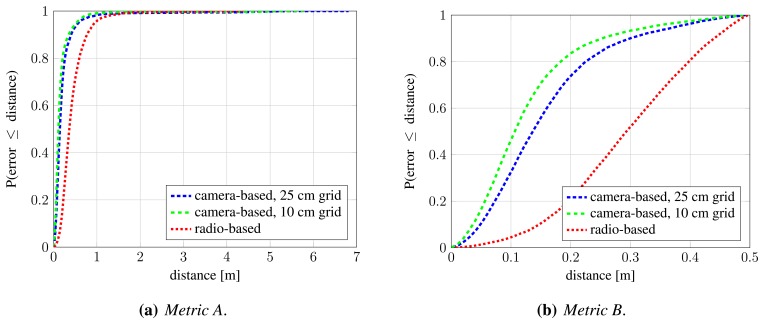
Comparison of camera-based and radio-based detection and localization: cumulative distribution function (CDF) of localization error under *Metric A* (**a**) and *Metric B* (**b**). The steeper the CDF curve and the sooner it reaches 1.0, the better. The operating point for camera-based system is *σ_pom_* = 0.005.

Figure 10 shows cumulative distribution function (CDF) of localization error for radio-based system and camera-based system in the chosen operating point, under *Metric A* and *Metric B*. Again, it can be seen that camera-based detections are much better localized.

It is worth noting that this is also the case under *Metric B*, which considers only detections within the specified threshold. Therefore, we can conclude that the radio-based system provides very reliable detection, with practically no false positive and negative detections, but with considerable localization error in a cluttered environment. On the other hand, computer-vision-based approach offers better localization, but the detection sometimes suffers from false positives and false negatives. These findings are consistent with those of our previous work [41].

### 6.3. Improvement of Anonymous Detection and Localization

**Figure 11.**
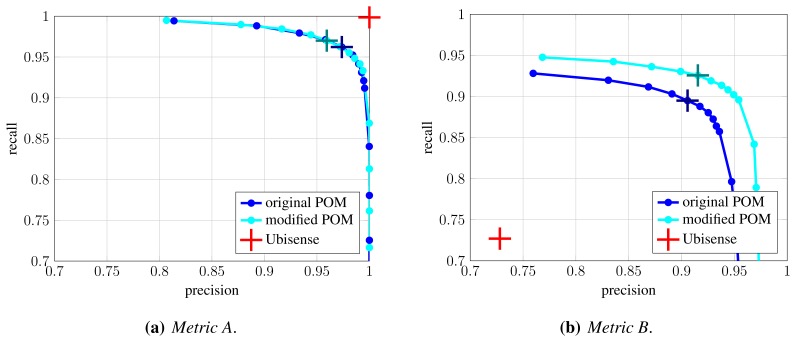
Evaluation of first fusion stage: precision and recall curves under *Metric A* (**a**) and *Metric B* (**b**). The colored curves represent precision-recall curves for original and modified POM as *σ_pom_* parameter is varied. In both cases, the occupancy map has resolution of 25 cm. The crosses on the curves denote the operating point *σ_pom_* = 0.005. The red cross denotes the result of radio-based localization system.

**Figure 12.**
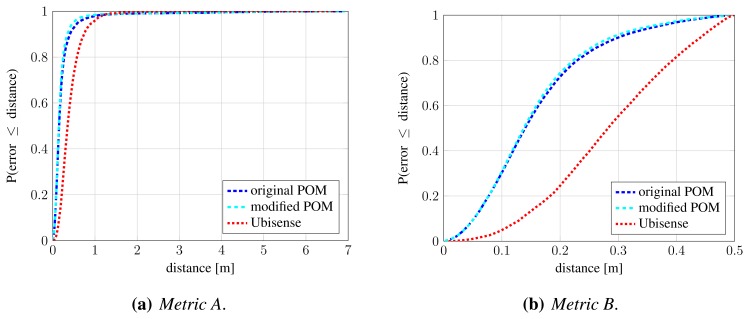
Evaluation of first fusion stage: cumulative distribution function (CDF) of localization error under *Metric A* (**a**) and *Metric B* (**b**). The steeper the CD curve and the sooner it reaches 1.0, the better. Occupancy map resolution is 25 cm and *σ_pom_* = 0.005.

**Figure 13.**
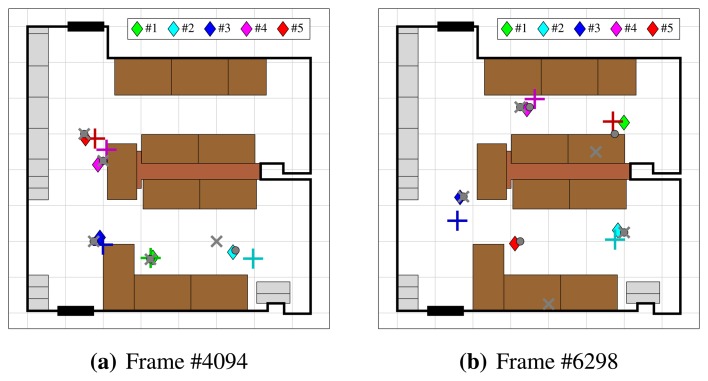
Results of first fusion stage: improved localization of Person #2 (**a**), and improved localization of Persons #1 and #5, with spurious additional detection for Person #4 (**b**). Note the poor localization of radio tag #5 and missing detection of radio tag #1. *Colored diamonds* denote ground-truth points, *colored crosses* denote radio-based detections, *gray X-marks* denote anonymous detections by original POM and *gray circles* denote anonymous detections obtained by modified POM.

Here, we evaluate the first stage of fusion, which aims to improve anonymous detection and localization (Section 4.1). We run the original and modified POM algorithm on the second portion of the dataset (frames 3721–7761), using occupancy map resolution of 25 cm. Evaluation is performed using methodology from Section 5.1. Figure 11 shows precision-recall curves as the *σ_pom_* parameter is varied. Under *Metric B* (Figure 11(b)), which characterizes system's localization performance, the shift of the data points on the curve indicates improvement in recall and slight improvement in precision. This means that the proposed combination of systems improves localization of anonymous detections, which is also reflected by slight increase in steepness of CDF curves for localization error, shown in Figure 12. An example is shown in Figure 13(a). However, under *Metric A* (Figure 11(a)), which characterizes detection performance, the shift of data points indicates somewhat lowered precision in individual operating points. The reason for this is that the proposed combination of systems sometimes results in two detections in adjacent cells. In cases, such as the one illustrated by Figure 13(b), this results in decreased precision under *Metric A*, but increased recall under *Metric B*. Multiple detections in adjacent cells could be handled by clustering of detections (which was done in [41]), however this would adversely affect situations when people come close together. Instead, we let them to be handled by the next stage of our fusion.

### 6.4. Tracking by Identification

Finally, we evaluate performance of the whole proposed tracking-by-identification system, which consists of KSP tracking step on top of improved anonymous detections that have been augmented with identities from radio tags (Section 4.2). We compare its performance to that of pure radio-based localization and identification, and that of state-of-the-art computer-vision-based identification-by-tracking system, which consists of KSP tracking on top of anonymous POM detections.

For construction of occupancy maps, we use 25 cm grid, and set operating point at *σ_pom_* = 0.005. As a result of identification by tracking, when KSP is used on top of anonymous POM detections, the tracker correctly returns five trajectories, to which we assign the identities of the individuals they were initialized on. During tracking by identification, we run KSP tracker separately on top of each sequence of identified detections, which results in five separate identified trajectories. We evaluate these trajectories and the identified detections from the radio-based system using methodology from Section 5.2.

**Figure 14.**
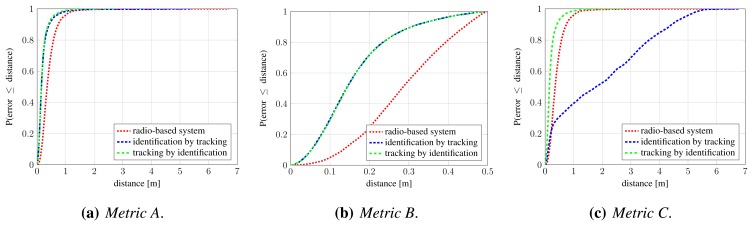
Evaluation of the proposed tracking-by-identification system: Cumulative Distribution Function (CDF) of localization error under *Metric A* (**a**), *Metric B* (**b**), and *Metric C* (**c**).

Figure 14 shows CDF of localization error under all three metrics, *Metric A*, *Metric B* and *Metric C*. Evaluating only anonymous detection and localization (*Metric A* and *Metric B*) results in practically identical curves for identification-by-tracking and tracking-by-identification, as shown in Figure 14(a) and 14(b), and is consistent with earlier findings that computer vision offers better localization than radio. However, it can be seen from Figure 14(c) that identification-by-tracking suffers from propagated identity switches, which manifest themselves as localization error. The radio-based system and the proposed tracking-by-identification system do not have such problems; their localization error CDF curves remain practically unchanged between Figure 14(a) and 14(c).

Propagated identity switches are also evident from confusion matrices in Table 4. For radio-based localization system (Table 4(a)), the lowered precision and recall are actually the result of poor localization and not identity switches. Most of the errors consist of phantom and missing detections, which are due to enforced threshold *T_d_* = 0.5 m. The rest of non-diagonal elements are from cases when for a person with a given tag, a different tag was localized closer to them than their actual tag was. In case of identification-by-tracking (Table 4(b)), the relatively high values of non-diagonal elements indicate propagated identity switches. There are also occurrences of missing and phantom detections, which are caused by longer periods of missing anonymous detections that serve as input to tracker. These cause the tracker to drift around, causing localization error, which, due to enforced threshold, is captured in missing and phantom detection statistics. Same problems occur with proposed tracking by identification approach (Table 4(c)), as it uses the same tracker. However, significantly lower values of non-diagonal elements indicate that identity switches occur only when people come close, and are not propagated as they disperse again. This confirms that the proposed combination of radio-based and computer-vision-based system successfully prevents propagation of identity switches.

**Figure 15.**
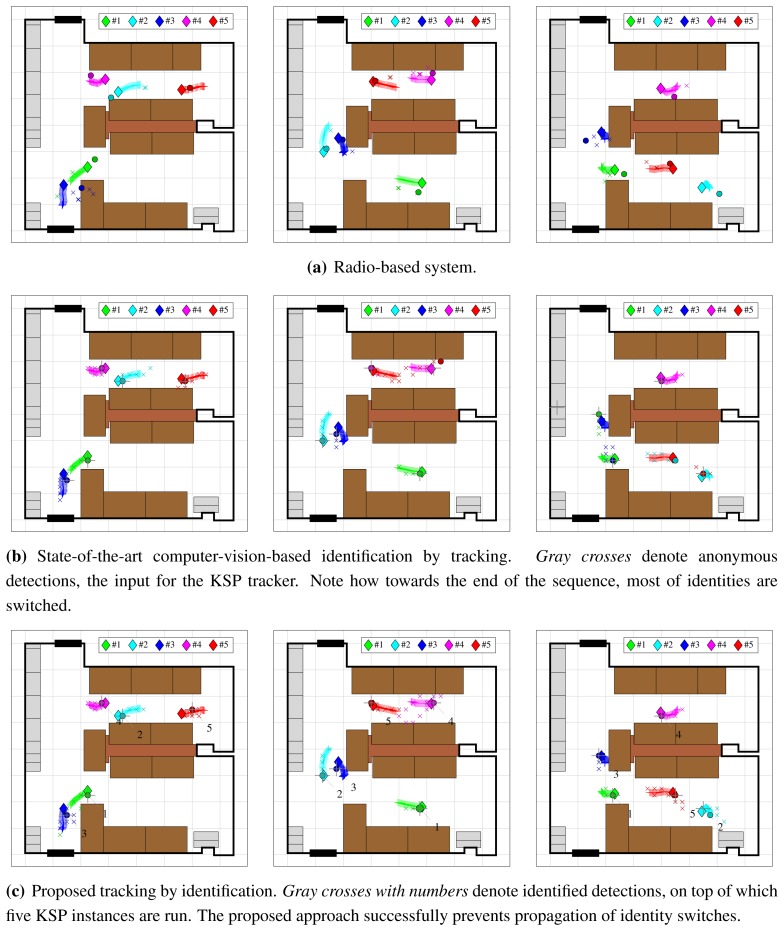
Visualization of tracking results for radio-based system (**a**), state-of-the-art computer-vision-based identification by tracking (**b**) and the proposed tracking by identification (**c**). *Colored diamonds* denote ground-truth annotations, while *colored circles* denote identified detections returned by the system. *Colored crosses* and *colored x-signs* are used to visualize 25-frame trails of ground-truth annotations and detections for better impression of their movement. For each system, three frames are shown (from left to right): #3751, #3830 and #7200.

For better illustration, we generated videos of tracking results, which can be found in the paper's supplementary material (http://mvg.fe.uni-lj.si/∼rokm/sensors_2012_supplementary/). Representative frames for radio-based detection, state-of-the-art identification by tracking and proposed tracking by identification are shown in Figure 15.

For the sake of completeness, we also performed experiment with 10 cm occupancy map. It should be noted that due to much denser grid (5,396 cells instead of 899), the problem that KSP solves becomes significantly more memory-intensive. For the used test sequence (4,040 frames), the memory consumption rose from 2.7 GB to 16.7 GB. The denser grid yields trajectories with less jitter, and as can be seen from confusion matrices in Table 5, less propagated identity switches in case of identification by tracking. However, they are still present, whereas the proposed tracking by identification successfully prevents them.

## 7. Conclusions

We presented a novel person localization system that performs tracking by identification, by combining a commercially available person detection and localization system based on Ultra-Wideband radio technology, and a state-of-the-art computer-vision based system for person detection and tracking with multiple calibrated cameras. The proposed system combines the best of both worlds: good localization offered by computer vision and strong identity information provided by the radio system. Therefore, compared with the radio-based system, the proposed solution offers much better localization, while at the same time addressing the problem of propagated identity switches that are present in the used state-of-the-art computer-vision based system.

Due to use of radio tags, the system is currently suitable for applications where wearing such is permissible; however, we expect that eventual miniaturization of radio tags will allow wider adoption also in various sports applications and that combination of radio-based and computer-vision based localization and identification will become more common due to the complementary nature of both technologies. Furthermore, the presented fusion scheme addresses a conceptual problem of combining a system whose detections are well-localized but not necessarily identified, and a system whose detections are reliably identified, but poorly localized, either because of difficult environment or due to physical limitations of the system. One can envision, for example, combining a computer-vision-based system with a coarse grid of RFID readers located in the floor tiles and passive RFID tags attached to individuals' shoes.

For the purpose of evaluating person detection and localization systems, we introduced a comprehensive metric and evaluation methodology that, using assignments constructed with different cost functions, captures different aspects of a system, namely detection, localization and identification performance. We demonstrated the use of proposed methodology first by comparing detection and localization performance of radio-based and computer-vision-based system, and then by full performance evaluation of both systems and their proposed combination.

Experimental results show the advantage of tracking-by-identification over identification-by-tracking approaches that are commonly found in the field of computer vision. Therefore, our future work will mostly focus on further pursuing this idea, even in purely computer-vision-based approaches, where individuals' identities would be determined from many weakly discriminative visual cues. Such development would on one hand allow completely unobtrusive tracking-by-identification and on the other hand further improve the presented system.

## Figures and Tables

**Table 1.** Summarized comparison of radio- and camera-based detection and localization, based on our previous work [41] and experimental results from Section 6.2.

	**Radio-based detection** (Section 3.1)	**Camera-based detection** (Section 3.2)
**False positive detections**	none	some
**False negative detections**	practically none	some
**Localization of detections**	poor	very good
**Identity information**	strong (tag ID)	none
**Data capture frequency [Hz]**	4.6 (five people)	20 (cameras' framerate)

**Table 2.** Time required for constructing an occupancy map with resolution 25 cm (899 locations) and 10 cm (5,396 locations, respectively) from four views as the images are scaled down. Measurements were performed on computer with Intel® Core™ i7 950 CPU, clocked at 3.07 GHz, and 18 GB RAM, using single-threaded POM implementation.

**Image size**	Processing time [s]	

	25 cm grid	10 cm grid

2,048 × 1,536	6.37	16.77
1,024 × 768	1.54	4.10
512 × 384	0.34	1.07
256 × 192	0.08	0.31
128 × 96	0.03	0.15

**Table 3.** Comparison of camera-based and radio-based detection and localization: precision, recall and localization error for several operating points (*σ_pom_* values) of camera-based system and a single operating point of radio-based system, evaluated under *Metric A* and *Metric B*.

**System**			**Metric A**			**Metric B**		

			precision	recall	localization error mean ± std [m]	precision	recall	localization error mean ± std [m]

**radio-based**			1.00	1.00	0.44 ± 0.34	0.71	0.71	0.30 ±0.11

**camera-based**, at specified *σ_pom_* values	25 cm grid	0.003	0.95	0.96	0.22 ± 0.37	0.90	0.90	0.16 ±0.10
		0.005	0.97	0.94	0.21 ±0.33	0.92	0.89	0.16 ±0.10
		0.007	0.98	0.93	0.21 ±0.31	0.93	0.89	0.16 ±0.10
		0.009	0.99	0.92	0.20 ± 0.30	0.94	0.88	0.16 ±0.10
		0.010	0.99	0.92	0.20 ± 0.30	0.94	0.88	0.16 ±0.10
		0.020	1.00	0.87	0.19 ±0.29	0.96	0.84	0.16 ±0.10

	10 cm grid	0.003	0.97	0.95	0.19 ±0.36	0.92	0.90	0.14 ±0.10
		0.005	0.99	0.94	0.18 ±0.32	0.94	0.89	0.13 ±0.09
		0.007	0.99	0.90	0.16 ±0.19	0.95	0.86	0.13 ±0.09
		0.009	1.00	0.86	0.15 ±0.15	0.96	0.83	0.13 ±0.09
		0.010	1.00	0.84	0.15 ±0.15	0.97	0.81	0.13 ±0.09
		0.020	1.00	0.27	0.13 ±0.10	0.98	0.27	0.12 ±0.09

**Table 4.** Evaluation of the proposed tracking-by-identification system: comparison with results of radio-based localization and state-of-the-art computer-vision-based identification-by-tracking approach. As described in Section 5.2, we construct confusion matrix using *Metric B* and comparing identities of assigned points. The rows of confusion matrix sum up to number of all ground-truth points with corresponding identities, whereas columns sum up to number of all detections with corresponding identities. Per-person precision and recall are obtained by taking diagonal value and dividing it by row or column sum, respectively.

		**DETECTIONS**					**Missing**	**Precision**	**Recall**

		**#1**	**#2**	**#3**	**#4**	**#5**			
**PERSON**	**#1**	**3092**	43	101	0	37	768	0.77	0.77
	**#2**	61	**3049**	12	0	0	919	0.76	0.75
	**#3**	15	9	**2048**	0	26	1943	0.51	0.51
	**#4**	0	0	0	**3071**	8	962	0.76	0.76
	**#5**	30	8	51	5	**3023**	924	0.75	0.75

**Phantom**		839	928	1811	965	943		**0.71**	**0.71**

(**a**) Radio-based system (Ubisense).									

		**DETECTIONS**					**Missing**	**Precision**	**Recall**

		**#1**	**#2**	**#3**	**#4**	**#5**			
**PERSON**	**#1**	**672**	238	1139	564	899	529	0.17	0.17
	**#2**	1186	**981**	1049	299	254	272	0.24	0.24
	**#3**	971	998	**1415**	385	164	108	0.35	0.35
	**#4**	0	324	0	**1902**	1435	380	0.47	0.47
	**#5**	924	1338	109	606	**851**	213	0.21	0.21

**Phantom**		288	162	329	285	438		**0.29**	**0.29**

(**b**) State-of-the-art *identification by tracking*: tracking on top of anonymous detections.									

		**DETECTIONS**					**Missing**	**Precision**	**Recall**

		**#1**	**#2**	**#3**	**#4**	**#5**			
**PERSON**	**#1**	**3565**	24	31	2	22	397	0.88	0.88
	**#2**	76	**3703**	12	0	26	224	0.92	0.92
	**#3**	77	36	**3661**	0	31	236	0.91	0.91
	**#4**	14	0	0	**3822**	1	204	0.95	0.95
	**#5**	24	17	39	7	**3784**	170	0.94	0.94

**Phantom**		285	261	298	210	177		**0.92**	**0.92**

(**c**) Proposed *tracking by identification*: tracking on top of identified detections.									

**Table 5.** Comparison of proposed tracking by identification with state-of-the-art identification by tracking, with occupancy map resolution being 10 cm.

		**DETECTIONS**					**Missing**	**Precision**	**Recall**

		**#1**	**#2**	**#3**	**#4**	**#5**			
**PERSON**	**#1**	**2192**	1144	13	0	122	570	0.54	0.54
	**#2**	586	**794**	2451	0	8	202	0.20	0.20
	**#3**	890	1548	**1502**	0	0	101	0.37	0.37
	**#4**	11	0	0	**3644**	31	355	0.90	0.90
	**#5**	15	129	0	6	**3765**	126	0.93	0.93

**Phantom**		347	426	75	391	115		**0.59**	**0.59**

(**a**) State-of-the-art *identification by tracking*: tracking on top of anonymous detections.									

		**DETECTIONS**					**Missing**	**Precision**	**Recall**

		**#1**	**#2**	**#3**	**#4**	**#5**			
**PERSON**	**#1**	**3714**	34	24	0	15	254	0.92	0.92
	**#2**	96	**3720**	10	0	0	215	0.92	0.92
	**#3**	26	13	**3879**	0	5	118	0.96	0.96
	**#4**	2	0	0	**3830**	9	200	0.95	0.95
	**#5**	4	1	5	8	**3940**	83	0.98	0.98

**Phantom**		199	273	123	203	72		**0.94**	**0.94**

(**b**) Proposed *tracking by identification*: tracking on top of identified detections.									
